# Micro-nano oxygenated irrigation improves the yield and quality of greenhouse cucumbers under-film drip irrigation

**DOI:** 10.1038/s41598-023-45121-3

**Published:** 2023-11-09

**Authors:** Zan Ouyang, Juncang Tian, Xinfang Yan, Zhenfeng Yang

**Affiliations:** 1 College of Water Resource and Civil Engineering, Hunan Agricultural University, Changsha, 410128 Hunan China; 2 School of Civil and Hydraulic Engineering, Ningxia University, No. 539 Helanshan West Road, Yinchuan, 750021 Ningxia China; 3 Engineering Technology Research Center of Water-Saving Irrigation and Water Resource Regulation in Ningxia, Ningxia University, Yinchuan, 750021 Ningxia China; 4 Engineering Research Center for Efficient Utilization of Modern Agricultural Water Resources in Arid Regions, Ministry of Education, Ningxia University, Yinchuan, 750021 Ningxia China

**Keywords:** Hydrology, Environmental impact

## Abstract

To study the influence mechanism of micro-nano oxygenated irrigation (MNOI) on greenhouse fruit cucumber in arid and semi-arid cold regions, the yield and quality of greenhouse fruit cucumber were evaluated and verified based on 2 years of observation data. Taking fruit cucumber in Ningxia solar greenhouse as the research object, three dissolved oxygen (DO) levels of MNOI (DO; 6, 7.5, and 9 mg L^−1^, O1, O2, and O3, respectively) and non-oxygenated irrigation (CK, 4 mg L^−1^) were set up as the control treatment. Through comparative design, the influence mechanism of different levels of aerobic irrigation on the yield and quality of greenhouse fruit cucumber was studied. The main indicators of fruit cucumber yield and quality increased with dissolved oxygen in irrigation water from 4 to 9 mg L^−1^. In spring–summer (autumn–winter), compared with CK, the leaf area index (LAI) and net photosynthetic rate (A) increased by 28.83% (28.77%) and 44.90% (35.00%), respectively, and Vitamin C, soluble protein, soluble sugar, soluble solids and total acid content increased by 100.00% (51.88%), 37.78% (61.11%), 34.17% (54.17%), 37.07% (78.72%) and 26.92% (30.67%) respectively, while nitrate content decreased by 44.88% (51.15%), and dry matter accumulation (DMA), soil respiration rate (SRR), microbial carbon (MC), and microbial nitrogen (MN) increased by 49.81% (127.25%), 55.22% (110.34%), 117.50% (90.91%) and 70.37% (74.42%) respectively, and yield, irrigation water use efficiency (IWUE) and soil oxygen content (SO) increased by 22.47% (28.04%), 22.39% (28.05%) and 33.21% (35.33%) respectively. A model of DO in irrigation water and SO was established and the applicability of the model was verified with an average relative error of 2% (less than 5%). MNOI increased SO and soil enzyme activity, enriched soil microorganisms, improved soil microenvironment, promoted water nutrient uptake and growth of root system, increased chlorophyll, photosynthesis and DMA, which improved fruit cucumber yield and quality, and the better DO concentration in irrigation water is 9 mg L^−1^. The research results provide theoretical support for regulating soil water, fertilizer and air environment, and at the same time, provide feasible ways to improve the quality and efficiency of crops in arid and semi-arid cold regions.

## Introduction

The fruit cucumber is a kind of cucumber (*Cucumis sativus* L.), also known as the thornless cucumber. With the continuous improvement of people’s living standards and the change of consumption concept, people have put forward higher requirements for the yield and fruit quality of the fruit cucumber. The fruit yellow melon with good taste and strong flavor is widely welcomed by consumers, therefore, it is incumbent on many scientific researchers to improve the yield and fruit quality of fruit cucumbers. The fruit cucumber has broad market prospects, but they are facing double challenges of water shortage and low temperature, when planted in the arid and semi-arid cold regions of Ningxia, China, the average annual precipitation of less than 200 mm, which poses a threat to the irrigation of local facility agriculture, and more importantly, long-term drip irrigation and flood irrigation lead to hypoxia in the root zone of crops. The study found that the oxygen diffusion rate in the central area of the drip irrigation emitter is the lowest^1^, under the condition of hypoxia, a series of physiological and biochemical changes occur in plants, such as decreased water absorption and mineral element absorption^2^, inhibition of photosynthesis^2,3^, changes in respiratory metabolic pathways^4^, imbalance of energy supply and demand, lack of energy materials, and inhibition of plant growth^5^. How to change the root-soil gas environment in drip irrigation is of great significance.

Oxygenated irrigation, aerated irrigation, and other oxygenation measures can improve soil ventilation^6–9^, which are widely used by many researchers to study crop quality and efficiency improvement. On the one hand, oxygenated irrigation (OI) can improve crop yield^10–14^. The appropriate O_2_ content in the soil tillage layer is more conducive to root growth at the cucumber seedling stage^15^. Micro-nano air bubble water can significantly improve cucumber yield, irrigation water use efficiency, and fruit quality (*P* < *0.05*), Cucumber yield, irrigation water use efficiency, vitamin C, and soluble sugar increased by 22.1%, 22.1%, 16.7%, and 19.4% respectively^16^. Du et al.^17^ showed that compared with non-oxygenated irrigation drip irrigation, OI is conducive to improving crop yield and water use efficiency (with an average increase of 19.3% and 17.9% respectively). On the other hand, aerated irrigation can also improve crop quality. Under hypoxia stress, the fruit will ripen early, thus reducing the content of lycopene and vitamin C in the fruit^18^. The oxygen respiration of the root system is inhibited, affecting fruit development^19^. However, aerobic drip irrigation can significantly promote the growth of tomatoes, increase their yield^20^, and improve their quality^9,21,22^. At the same time, aerobic irrigation can also effectively improve crop water use efficiency^23–25^.

Aerated irrigation has also been proven to improve the yield and quality of tomatoes^7,24,26–28^, potatoes^29^, pumpkins^30^, melons^31^, rice^32–34^, and other crops. In addition, ventilation effectively controls the release of mineral elements in the soil, significantly affects the absorption of mineral elements by crops, promotes the exchange of heat between the soil and the atmosphere, and effectively maintains the uniform soil temperature^35^, and promotes soil microorganisms and soil respiration^36,37^. It can also make the crop root system, microbial activity, and mineral transformation in the underground drip irrigation area in the best state^23^.

However, in the arid and semi-arid cold regions of Ningxia, the effect mechanism of submembrane drip irrigation and oxygenation on greenhouse fruit cucumbers is still unclear, and MNOI is widely used in crop irrigation because of its good aerobic effect^7,38–40^. Based on previous studies^8,41–43^, this study is mainly aimed at the hypoxia problem caused by long-term drip irrigation and flood irrigation in the root zone of greenhouse fruit cucumber, aiming to regulate the gas environment in the root zone of greenhouse crops by MNOI, to reveal the response mechanism of the yield and quality of greenhouse fruit cucumber to MNOI, a new feasible way for improving the quality and efficiency of greenhouse fruit cucumber in semi-arid and cold regions was provided.

## Materials and methods

### Experimental site

**Figure 1:**
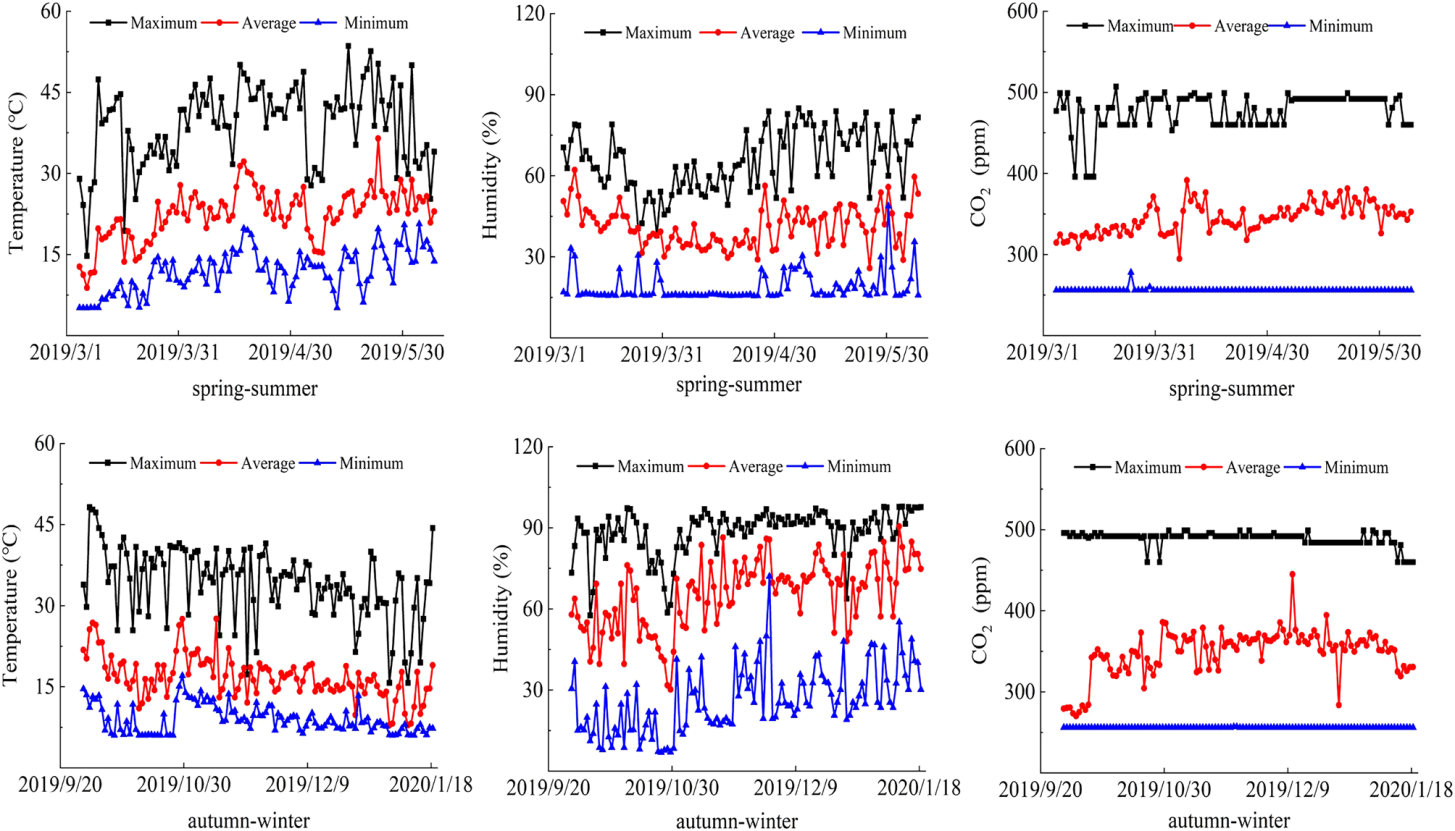
Changes in greenhouse air temperature, humidity, and CO_2_.

The study area is located in Xinrong Village Experimental Area, Helan County, Yinchuan (38°30′ N, 106°07′ E), Ningxia, with a temperate continental climate and an altitude of 1111.5 m, an average annual temperature of 10.7 °C, the maximum temperature of 31.5 °C, minimum temperature of − 11.1 °C, annual precipitation of 227.4 mm, annual evaporation of 1288 mm, and total annual sunshine hours of 2785 h. Average greenhouse temperature in spring–summer (autumn–winter) 9.0–34.4 ℃ (11.9–38.8 ℃), average humidity 26.4–88.7% (18.8–65.5%), average CO_2_ concentration 256.0–488.9 ppm (477.6–256.3 ppm), meteorological data were obtained from small weather stations set up inside and outside the greenhouse (Fig. 1).

The test soil is a sandy loam, the soil bulk density is 1.413 g cm^−3^, and the field capacity is 18.88%. Because the local groundwater is brackish water (salinity 2895 mg L^−1^, Na^+^ 1400 mg L^−1^, Cl^−^ 599 mg L^−1^, EC 385 μS cm^−1^, pH 8.08), direct irrigation is not beneficial to the growth of crops, the experimental greenhouse (75 m long and 7 m wide) was installed with reverse osmosis (RO) equipment developed by Ningxia University water saving irrigation innovation team and used for many years, this study aimed to desalinate the underground brackish water to meet the irrigation requirements of greenhouse experiment. The desalinated water was used for irrigation in this experiment (salinity 447 mg L^−1^, Na^+^ 63 mg L^−1^, Cl^−^ 186 mg L^−1^, EC 301 μS cm^−1^, pH 6.88), and the pH of 0–20 cm soil in the greenhouse was 8.65, the total salt content was 0.53 g kg^−1^, the organic matter content was 17.46 g kg^−1^, the alkali-hydrolyzed nitrogen was 67.16 mg kg^−1^, the available phosphorus was 96.99 mg kg^−1^, the available potassium was 402.50 mg kg^−1^.

### Experimental design

A comparative experimental design was adopted. Since the dissolved oxygen (DO) in the irrigation water was 4 mg L^−1^ (18–20 ℃) in its natural state under this experimental condition, and the saturated DO in the irrigation water was about 10 mg L^−1^ after the application of oxygenation measures, three levels of DO in the irrigation water of O1 (6 mg L^−1^), O2 (7.5 mg L^−1^), O3 (9 mg L^−1^) and the control treatment without oxygenation (CK, 4 mg L^−1^) were set, and three replications were set for each treatment, for a total of 12 treatments. The aim was to study the effect of different oxygenated irrigation on the yield and quality of greenhouse fruit cucumber, to reveal the response mechanism of greenhouse fruit cucumber yield and quality to oxygenated irrigation, and to propose the optimal level of oxygenated irrigation for the study area.

### Experimental implementation

Micro-nano oxygenation irrigation (MNOI) of greenhouse fruit cucumbers was carried out in 2 seasons of experiments, respectively in spring–summer (March 5, 2019–June 5, 2019, 93 days) and autumn–winter (September 28, 2019–January 18, 2020, 115 days), the test fruit cucumber (*Cucumis sativus* L.) variety was “Jasper No. 3”, the fruit cucumber (*Cucumis sativus* L.) selected for this study were provided by the Ningxia Tianyuan Seed Industry Co., Ltd., China. With three leaves at the time of transplanting, the experimental plot area was 7.7 m^2^ (length 5.5 m, width 1.4 m), with three replications of the same treatment. Each plot area is planted in two rows (plant spacing 0.45 m, row spacing 0.30 m) with a planting density of 31,185 plants hm^−2^, and two parallel inlaid drip irrigation strips were arranged in each plot, the distance between the two strips was 0.40 m, the diameter of the pipe was 16 mm, the distance between the dripper was 0.15 m, and the flow rate of the dripper was 2 L h^−1^.

During the whole experiment period, 27 times of irrigation (including 9 times of irrigation in the slow seedling stage) were carried out, and the irrigation quota was 3375 m^3^ hm^−2^. According to the method of nutrient balance to determine the amount of top dressing, the principle is to determine the amount of top dressing according to the difference between the amount of fertilizer needed for target yield and the amount of fertilizer available in the soil. The amount of top dressing for fruit and cucumber is N: 105 kg hm^−2^, P_2_O_5_: 30 kg hm^−2^, K_2_O: 75 kg hm^−2^, five times of top dressing, the types of fertilizer used for drip irrigation are urea (N 46%), diammonium hydrogen phosphate (N 16%, P_2_O_5_ 44%) and potassium sulfate (K_2_O 22%), of which urea is 204.55 kg hm^−2^, diammonium hydrogen phosphate 68.18 kg hm^−2^, potassium sulfate 340.91 kg hm^−2^, topdressing was carried out in the critical period of fertilizer requirement for fruit cucumber. Micro-nano aeration equipment (XZCP-K, Yunnan Xiazhichun Co., Ltd., China) was used to oxygenate the irrigation water, and the dissolved oxygen of the irrigation water was increased to the corresponding level and transported to the root area of fruit cucumbers through the drip irrigation system, to realize the comprehensive regulation of the water-fertilizer-gas environment in the root area of fruit cucumbers, and the number of oxygenated irrigation times of fruit cucumbers during the whole growth period was 10 times.

### Measurement items and methods

The team's self-developed reverse osmosis water treatment system and intelligent irrigation equipment were used for irrigation. The irrigation water was recorded by an electronic flow meter, and the dissolved oxygen analyzer (JPB-607A, INESA Scientific Instrument Co., Ltd., China) was used to measure the DO in the irrigation water after the irrigation water was micro-nano-oxygenated.

After transplanting, after the seedlings have passed the slow seedling stage (the end of the seedling stage), 3 plant markers with normal growth are randomly selected in each treatment, and the same plant is measured each time in the future. Plant height, stem thickness, leaf area index (LAI), and chlorophyll were recorded every 10 days, among which, the plant height was measured from the base of the stem to the top by a steel tape measure, the stem thickness was measured by a digital vernier caliper to measure the stem diameter of the main vine base, the LAI was measured by a canopy analyzer (LAI-2200 C, LI-COR, USA), and the chlorophyll was measured by a chlorophyll meter (SPAD-502, Konica Minolta, Japan), and the final yield was the sum of each pick. The net photosynthetic rate (A, μmol m^−2^ s^−1^), stomatal conductance (gsw, mol m^−2^ s^−1^), intercellular CO_2_ concentration (Ci, μmol mol^−1^), transpiration rate (E, mmol m^−2^ s^−1^), leaf temperature (Tleaf, °C) of fruit cucumber leaves were measured by a portable photosynthesis measurement system (LI-6800, LI-COR Company), and repeated 3 times per treatment, photosynthetic measurements of fruit cucumber leaves were performed in fine weather (8:00, 10:00, 12:00, 14:00, 16:00) once per growth period. Fruit vitamin C, soluble total sugar, total acid, and soluble solids of fruit cucumber were detected by Ouyang et al.^7^, the soluble protein was determined according to Coomassie brilliant blue G-250 staining method, and nitrate was determined by ion chromatography (GB 5009.33-2016).

After complete harvesting, all above-ground parts of each treatment plant were mowed, and at the same time, a pit of about 40 cm in diameter and 40 cm in depth was dug at the center of the stem to obtain the root system, carefully shaking off the soil between the roots and picking up the residual roots to rinse the soil slowly with water, placing the roots and soil on a 100 mesh steel sieve during rinsing to minimize root loss. The roots were washed and placed in an oven with the stems, and left at 105 °C for 15 min, dried at 75 °C to a constant weight, and weighed.

The soil was taken before the test to determine the initial nutrients according to the “Methods of Agricultural Chemical Analysis of Soil”. Soil bacteria, fungi, actinomycetes, soil microbial carbon (MC), soil microbial nitrogen (MN), and soil urease, alkaline phosphatase (AP), and catalase activities were measured in 0–20 cm fresh soil near the end of the experiment^7^. Soil water content, temperature, and conductivity were monitored in real-time using TEROS12 sensors (Meter Group, Inc. USA) with a sensor burial depth of 20 cm, soil oxygen was monitored in real-time using oxygen sensors, and soil respiration rate (SRR) was measured using ACE automatic soil respiration meter (ADC, UK) at 9:00–11:00 am in clear weather.

The water consumption (ET_c_) of fruit cucumbers during the test period was calculated by the water balance method^7^. The groundwater depth in the study area was 3–4 m, and the recharge of groundwater to fruit cucumber was negligible; there was no surface runoff and deep seepage because the drip head irrigation intensity was less than the soil infiltration rate; there was no rainfall because the experiment was conducted in the greenhouse. Irrigation water use efficiency (IWUE) = Y/I, y is yield (kg), I is irrigation quota (m^3^); Water use efficiency (WUE) = Y/ET_c_, Y is yield (kg), ET_c_ is water consumption (m^3^); The instantaneous water use efficiency of leaves (WUE_int_) = A/E, A is the net photosynthetic rate (μmol m^−2^ s^−1^), E is the transpiration rate (mmol m^−2^ s^−1^).

Since the air boundary layer in the solar greenhouse belongs to the non-neutral stable stratification, evaporation, and heat transfer still exist when the wind speed is zero. To avoid the contradiction between the calculated results and the actual situation when the wind speed is zero, the reference crop evapotranspiration (ET_0_) was calculated according to the Penman–monteith formula modified by Wang et al.^44^ and Shi et al.^45^, which was suitable for the greenhouse environment.

1 $$ET_{0} = \frac{{0.408\Delta (R_{n} - G) + \gamma \frac{{1713(e_{a} - e_{d} )}}{{T_{mean} + 273}}}}{\Delta + 1.64\gamma }$$

In the formula, ET_0_ is the reference crop evapotranspiration, mm d^−1^; Δ is the slope of the relationship between saturated water vapor pressure and temperature, kPa °C^−1^; R_n_ is the net radiation, MJ (m^2^ d)^−1^; G is soil heat flux, which is very small and usually ignored, MJ (m^2^ d)^−1^; γ is the hygrometer constant; T_mean_ is the average air temperature at 2 m in the greenhouse, °C; e_a_ is saturated water vapor pressure, kPa; e_d_ is measured water vapor pressure in the greenhouse, kPa.

2 $$ET_{{\text{c}}} = K_{c} ET_{0}$$

where, ET_c_ is the actual crop evapotranspiration (actual crop water consumption), mm d^−1^; K_c_ is the crop coefficient.

### Data processing

The data were sorted out by Excel 2021 software, Data Processing System 18.10 (Hangzhou Ruifeng Information Technology Co., Ltd., China) software was used for the analysis of extreme variance, analysis of variance, and Duncan’s multiple comparisons of the observed data of fruit cucumbers in the same season. Origin 2023 learning version software (OriginLab Corporation, USA) was used for drawing.

### Ethical statement

We ensured that the collection of plant material and experimental research and field studies on plants complied with relevant institutional, national, and international guidelines and legislation.

## Results and analysis

### Effects of MNOI on the growth of fruit cucumber

**Figure 2:**
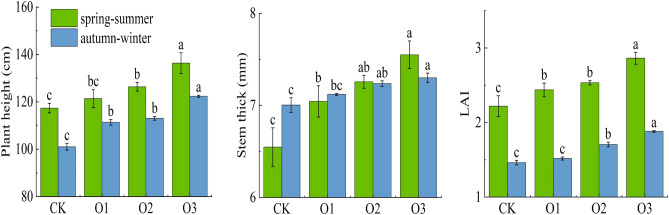
Changes of fruit cucumber plant growth. Different lowercase letters in the same season indicate significant differences among all treatments (P < 0.05). The following graphics represent the same meaning.

Figure 2 shows the effect of dissolved oxygen on plant height, stem diameter, and LAI. Data on plant height, stem thickness, and LAI were obtained from observations made for fruit cucumber at maturity. Plant height, stem thick and LAI increased with increasing oxygen concentration in spring–summer and autumn–winter (O3 > O2 > O1 > CK), and compared to CK treatment, plant height, stem thick and LAI increased by 16.20% (21.09%), 15.27% (4.29%), 28.83% (28.77%) in spring–summer (autumn–winter) O3 treatment, respectively.

The effects of DO on plant height, stem thickness and LAI were highly significant (P < 0.01). The effect of DO on growth indicators showed consistent patterns: plant height, stem thickness and leaf area index increased with increasing DO in irrigation water, indicating that increasing DO could promote the growth of fruit cucumber plants to some extent, mainly because oxygen-enhanced irrigation delivered irrigation water containing oxygen to the vicinity of the root system, which improved the gas environment in the root zone and significantly promoted the root length, density and weight, and enhanced It also enhanced the root system’s ability to absorb water and fertilize, further promoting root growth and development, thus increasing plant height, stem thickness and LAI to different degrees.

### Effects of MNOI on the photosynthesis of fruit cucumber

**Figure 3:**
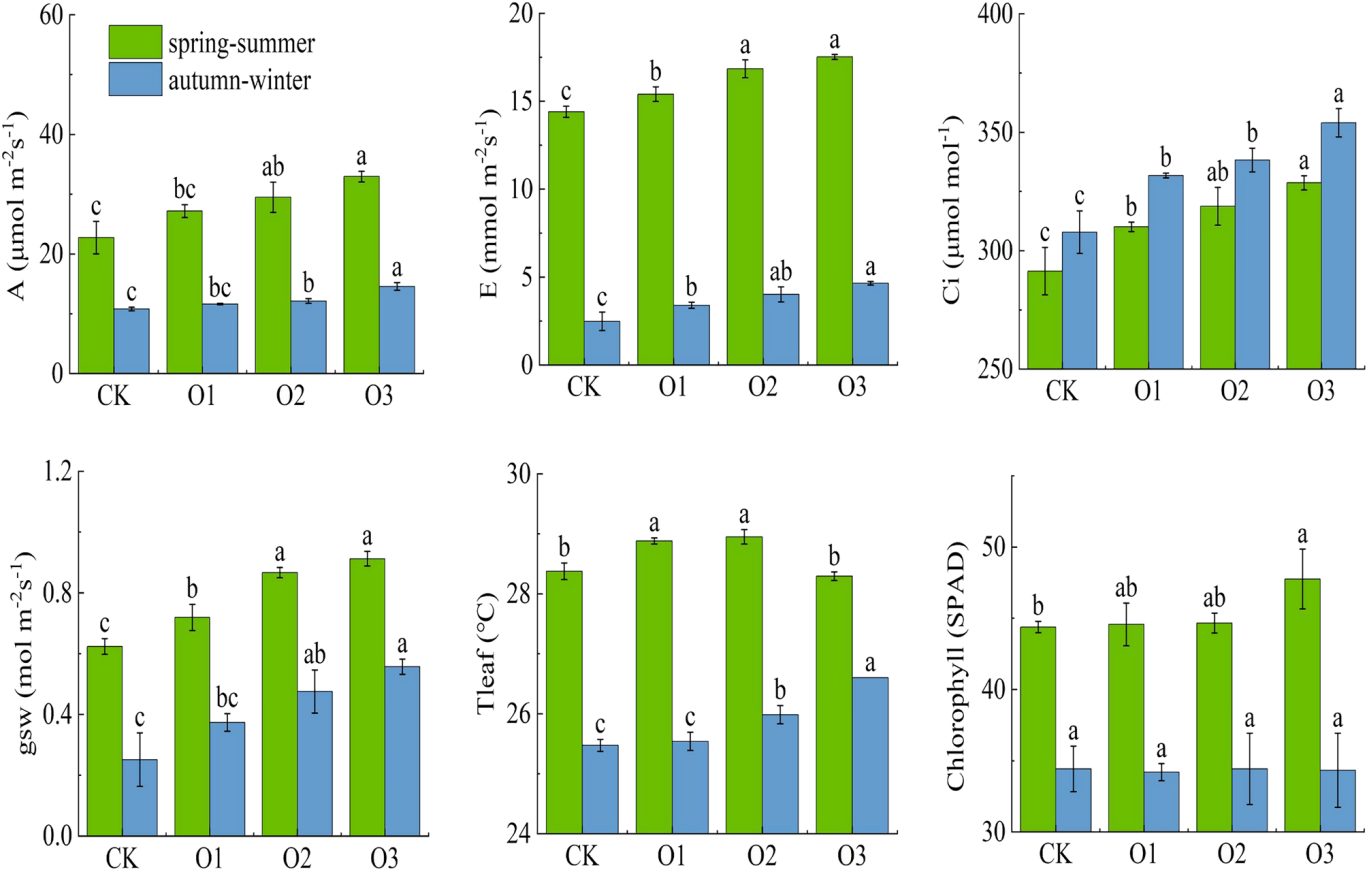
Changes of fruit cucumber plant photosynthetic.

Figure 3 represents the effect of DO in irrigation water on A, E, Ci, gsw, Tleaf, and chlorophyll. Photosynthetic characteristics were observed on May 30, 2019 (spring–summer) and November 15, 2019 (autumn–winter), and the specific time of observation was 9:00 a.m. to 10:00 a.m. The changes of photosynthetic characteristics indexes for each treatment in spring–summer and autumn–winter were: O3 > O2 > O1 > CK, compared with CK treatment, the A, E, Ci and gsw of O3 treatment in spring–summer (autumn–winter) increased by 44.90% (35.00%), 21.67% (86.75%), 13.06% (14.94%) and 46.15% (121.91%), respectively.

DO had a significant effect on A and gsw (P < 0.01), and the influence of DO on the main indicators of photosynthetic characteristics showed consistency: A, E, Ci and gsw all increased with the increase of DO in irrigation water, indicating that increasing DO can improve the photosynthesis of plants to a certain extent, which is conducive to the synthesis of organic matter and dry matter accumulation. It is mainly because low-oxygen stress often occurs in the root system during long-term drip irrigation or flood irrigation, resulting in a decrease in root vitality, a decrease in the absorption capacity of roots to soil water and nutrients, and a decrease in the energy supply of roots to the aboveground part, thereby limiting the absorption and utilization of nutrients by leaves, resulting in a decrease in leaf chlorophyll content, which in turn affects the photosynthesis of plants. In addition, root hypoxia increases the abscisic acid and ethanol content of fruit cucumber plants, causes a decrease in the stomatal opening, and reduces leaf photosynthesis and transpiration^46^. On the contrary, MNOI increased soil oxygen content, enhanced soil aeration, enhanced plant metabolic capacity, promoted the absorption and utilization of nutrients by plant roots, and improved the photosynthesis ability of leaves.

### Effects of MNOI on the fruit quality of fruit cucumber

**Figure 4:**
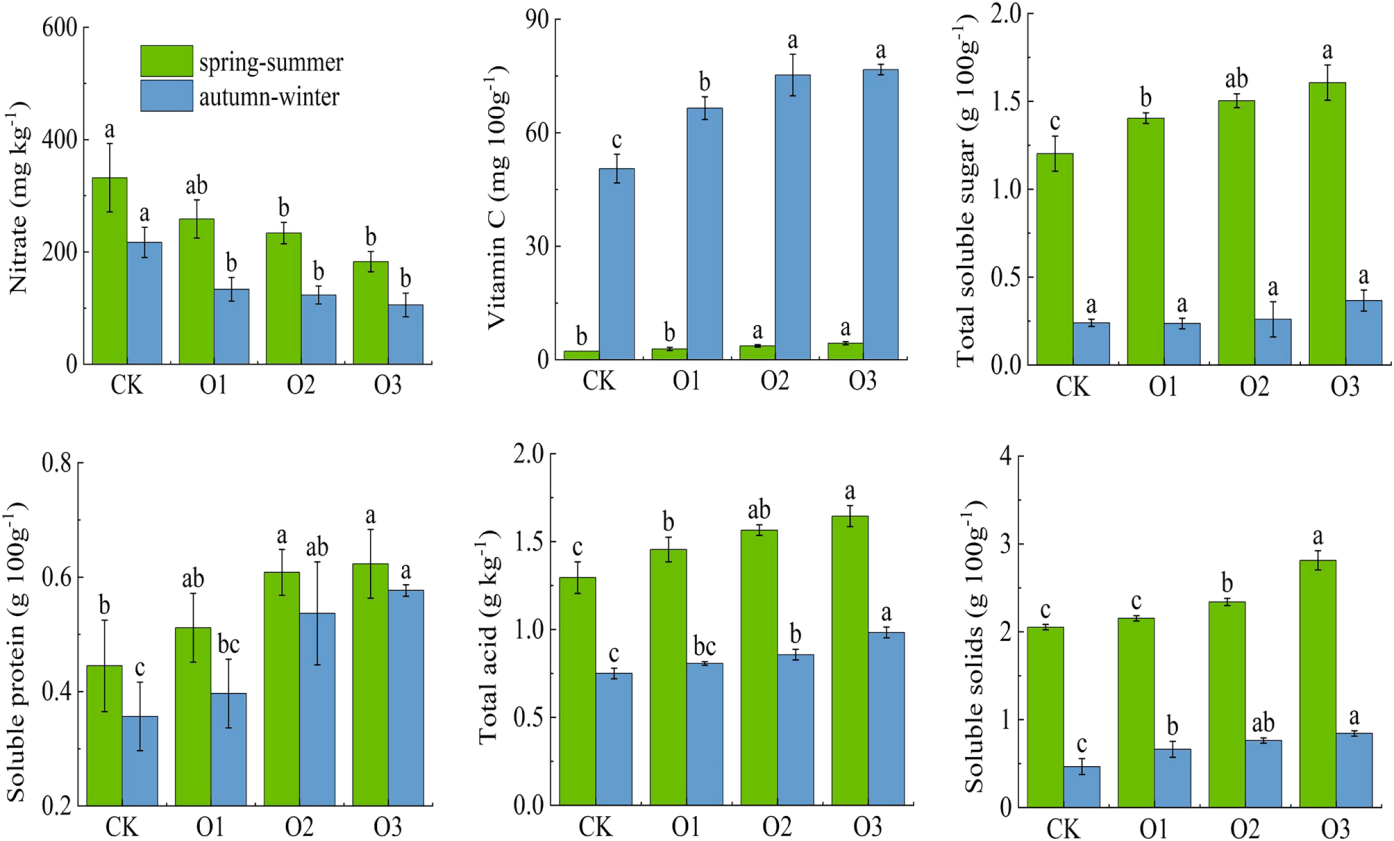
Changes of fruit cucumber fruit quality.

Figure 4 represents the effect of DO in irrigation water on the content of nitrate, vitamin C, soluble sugar, soluble protein, total acid and soluble solids in fruits. The main indexes in spring–summer and autumn–winter showed the same pattern of change (O3 > O2 > O1 > CK), while the trend of change in nitrate content was opposite to them (O3 < O2 < O1 < CK). Compared with the control treatment CK, in spring–summer (autumn–winter), fruit vitamin C, soluble protein, soluble sugar, soluble solids and total acid content of O3 increased by 100.00% (51.88%), 37.78% (61.11%), 34.17% (54.17%), 37.07% (78.72%) and 26.92% (30.67%), respectively, while nitrate content decreased by 44.88% (51.15%).

The effect of DO on fruit cucumber fruit vitamin C, soluble solids and total acid was highly significant (P < 0.01), and fruit vitamin C, soluble protein, soluble sugar, soluble solids and total acid increased with the increase of DO, while nitrate content decreased. It indicates that increasing DO in irrigation water can improve the quality of fruit cucumber fruits to some extent, and the factors affecting the quality of fruit cucumber are mainly divided into two aspects: endogenous factors mainly include variety and maturity, which may lead to differences in results in this study due to different fruit maturity at the time of harvesting, and exogenous factors are mainly the influence of soil, climate and facility management conditions of crop cultivation, and MNOI improved soil aeration, improved the root-soil growth environment, promoted the effective use of fertilizer by the root system, which in turn improved the fruit quality of fruit cucumber.

### Effects of MNOI on the plant biomass of fruit cucumber

**Figure 5:**
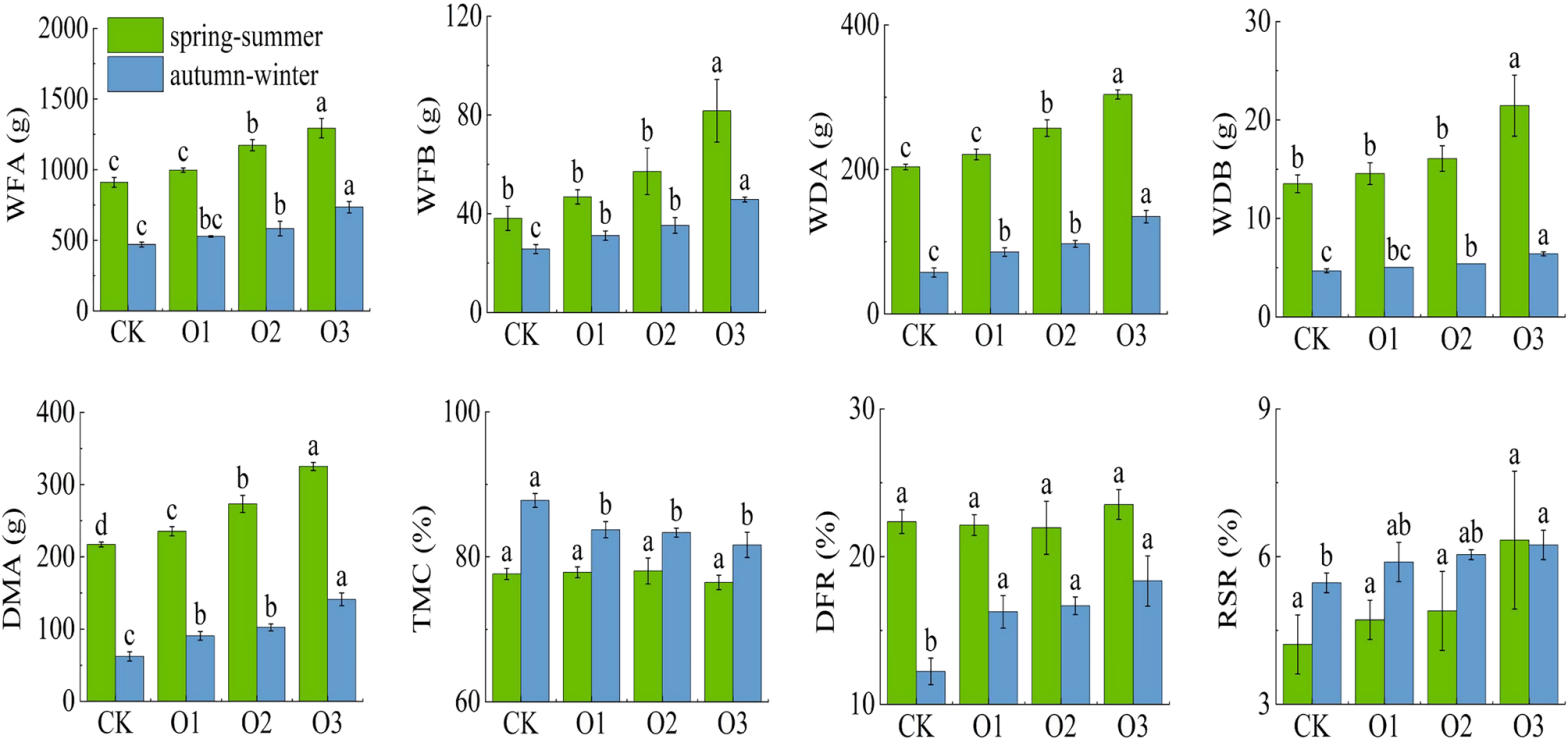
Changes of fruit cucumber plants biomass.

Figure 5 represents the effects of DO in irrigation water on fresh weight, dry weight, dry matter accumulation (DMA), dry-to-fresh ratio (DFR), root-to-shoot ratio (RSR) and tissue moisture content (TMC) of fruit cucumber plants. The distribution of DMA is an important part of crop growth and development, the accumulation increases with the growth process and is closely related to the crop yield, while the distribution of dry matter among different organs represents the growth and development of each part of the plant.

The effect of DO on above-ground fresh weight (WFA), below-ground fresh weight (WFB), above-ground dry weight (WDA), below-ground dry weight (WDB) and DMA was highly significant (P < 0.01), and WFA, WFB, WDA, WDB, DMA and RSR of plants in spring–summer and autumn–winter increased with dissolved oxygen (O3 > O2 > O1 > CK). Compared with the control treatment CK, the WFA, WFB, WDA, WDB and DMA of fruit cucumber increased by 42.90% (56.38%), 114.44% (134.67%), 49.21% (78.21%), 59.26% (36.17%) and 49.81% (127.25%). The DMA increased with the increase of DO, which indicated that the increase of DO could promote the growth of the plant and increase the DMA of fruit and cucumber plants. The main reason is that the increase of oxygen content in the soil after oxygen-enriched irrigation makes the respiration rate of the root increase, which improves the growth ability of the root system, therefore, it is more effective for the growth and biomass accumulation of fruit cucumber.

### Effects of MNOI on the yield and WUE of fruit cucumber

**Figure 6:**
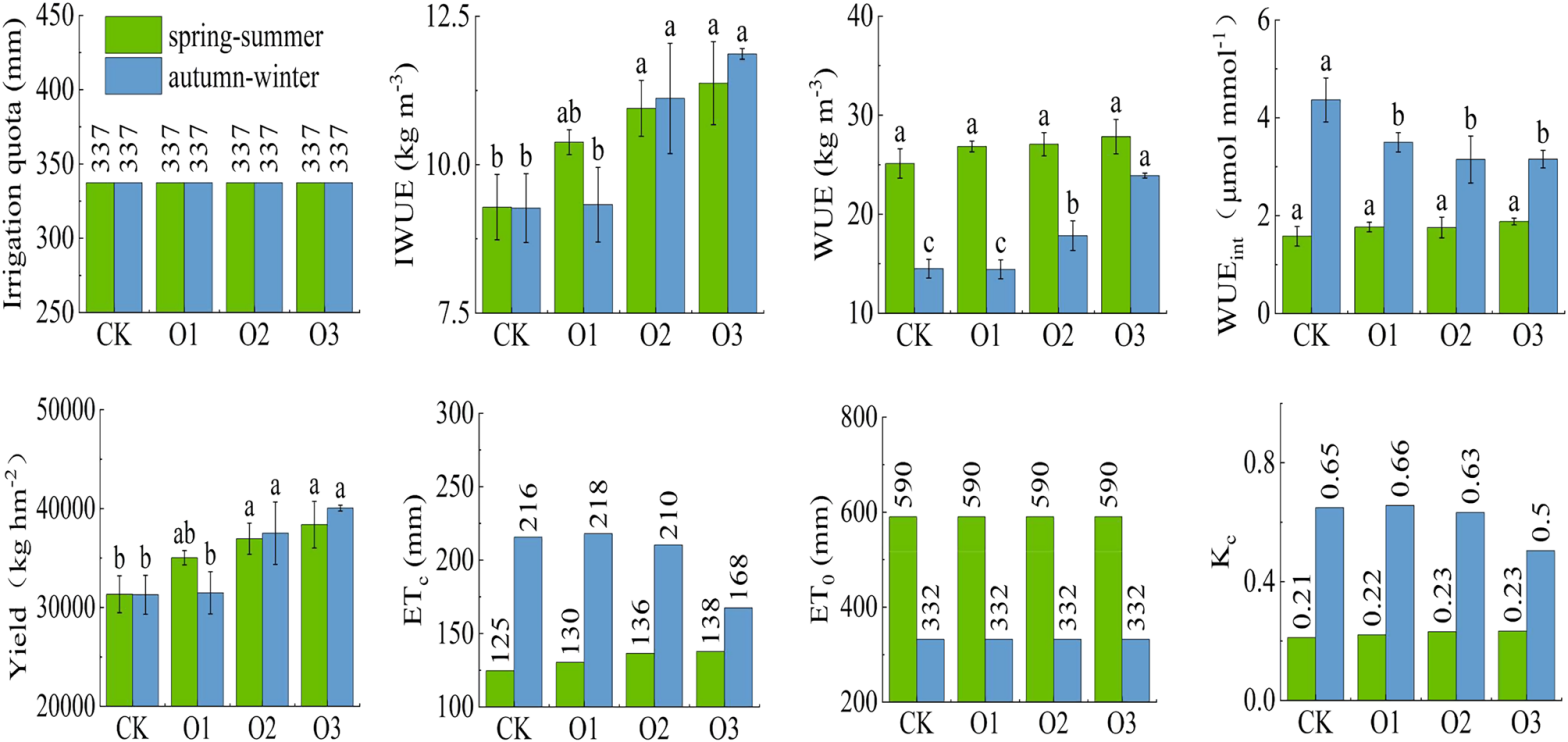
Changes of fruit cucumber yield and water use efficiency.

Figure 6 shows the effect of dissolved oxygen in irrigation water on fruit cucumber yield, IWUE and WUE. The yield, IWUE, WUE and WUE_int_ increased with increasing DO in irrigation water in both seasons (O3 > O2 > O1 > CK). Compared with CK, the yield, IWUE, WUE and WUE_int_ of O3 increased by 22.47% (28.04%), 22.39% (28.05%) and 18.99% (− 27.69%) in the spring–summer (autumn–winter), ET_0_ was 590.36 mm and 332.47 mm during spring–summer and fall-winter, respectively, and K_c_ increased by 10.53% for the spring–summer O3 treatment and decreased by 23.08% for the fall-winter O3. ET_0_ was 590.36 mm and 332.47 mm for the spring–summer and fall-winter test periods, respectively.

DO had significant effects on fruit cucumber yield, IWUE (P < 0.05) and WUE (P < 0.01), and yield, IWUE and WUE increased with the increase of DO in irrigation water, indicating that increasing DO in irrigation water not only improved fruit cucumber yield but also improved their IWUE and WUE. The yield of crops is affected by genetic factors and environmental factors, plant rhizosphere soil moisture, nutrients, salinity, gas, temperature and compactness are the main soil environmental factors affecting crop yield, the role of soil gas on plants is as important as soil water and nutrients, MNOI promotes crop photosynthetic rate and transpiration enhancement, water and nutrient absorption capacity is enhanced, plant growth is promoted, thereby improving WUE and achieving the purpose of improving quality and efficiency.

### Effects of MNOI on the soil microorganism and enzyme activity

Nitrogen-fixing bacteria in soil bacteria provide a source of nitrogen to the plant, and nitrifying bacteria avoid the accumulation of nitrite in the soil. Fungi break down cellulose, lignin and pectin and release nutrients, and the accumulation of mycelium improves the physical structure of the soil; however, fungi assimilate soil carbon and fix inorganic nutrients, a process that in turn competes with crops for nutrients^47^. Actinomycetes are major antibiotic-producing bacteria with good biocontrol effects and play an important role in adjusting the ecological balance of soil microorganisms. Soil peroxidase can promote the decomposition of hydrogen peroxide into water and oxygen, reduce the toxicity of hydrogen peroxide to crops, is closely related to soil respiration intensity and microbial activity, and is an important enzyme for soil fertility evaluation^48^. Soil urease, one of the important hydrolytic enzymes in soil, can hydrolyze urea applied to the soil to release ammonium, which plays an important role in the soil nitrogen cycle and reflects soil nitrogen supply capacity^49,50^. Soil phosphatase (Phosphatase) can hydrolyze organic phosphorus compounds in the soil into effective phosphorus that can be used by crops^51^.

**Figure 7:**
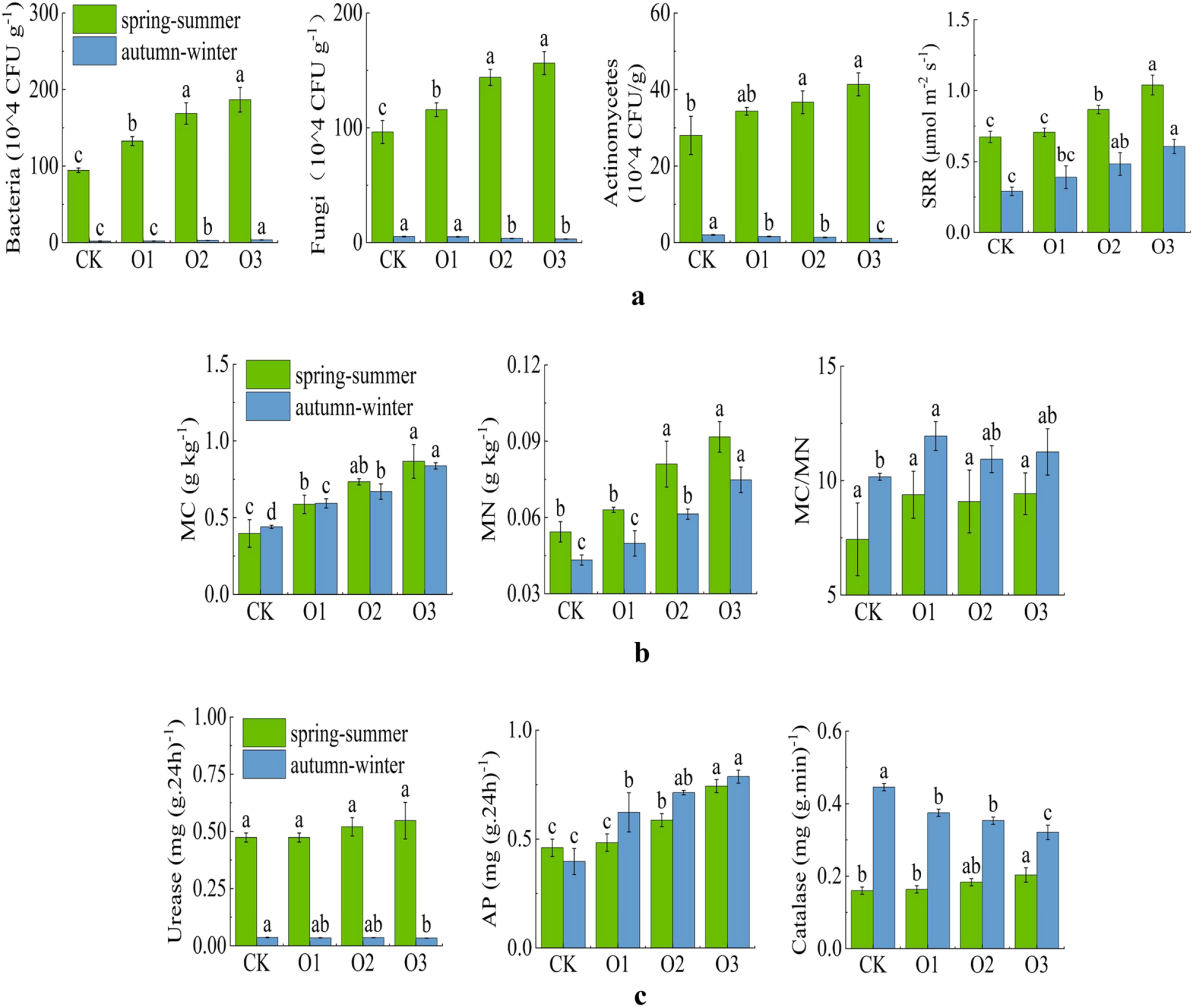
(**a**) Changes of soil bacterial, fungal, and SRR. (**b**) Changes of MC, MN and MC/MN. (**c**) Changes of soil urease, AP and catalase activities.

Figure 7a–c represents the effect of DO in irrigation water on soil microbial and enzyme activities. The effect of dissolved oxygen on soil bacteria, fungi, SRR, MC, MN, and AP of fruit cucumber was highly significant (P < 0.01) in both seasons, and the values of the main indicators of soil microbial and enzyme activities increased with the increase of DO in irrigation water (O3 > O2 > O1 > CK), in which, compared with the control treatment, soil bacteria in the spring–summer (autumn–winter) O3 treatment, fungi, and actinomycetes increased by 98.94% (94.44%), 62.50% (-38.46%), and 46.43% (-50.00%), respectively, SRR, MC and MN increased by 55.22% (110.34%), 117.50% (90.91%), and 70.37% (74.42%), respectively, and urease, AP and catalase increased by 17.02% (-5.61%), 60.87% (97.50%), and 25.00% (− 28.89%), respectively. It shows that increasing DO in irrigation water can increase soil microbial load as well as SRR to some extent.

This is mainly because MNOI can effectively relieve soil hypoxic stress, increase soil air conductivity, improve soil oxygen environment^52^, smooth aerobic respiration of the root system, improve plant WUE^29^, secure soil microbial activity and improve soil enzyme activity. Hydrogen peroxidase is widely present in soil and organisms, and MNOI increases the activity of hydrogen peroxidase, which promotes the decomposition of hydrogen peroxide and reduces the damage of hydrogen peroxide to plants. Oxygenated irrigation improves soil urease activity, which can hydrolyze urea applied to the soil and release ammonium, which can provide more effective nitrogen for plants, which is important to promote soil nitrogen cycle, improve soil fertility, and ensure normal plant growth^53^. Meanwhile, MNOI increases soil phosphatase activity, which can enhance the hydrolysis of catalytic phospholipids or phosphate intoxication, thus enhancing the conversion and utilization of soil organic phosphorus and improving the soil microbial environment.

### Effects of MNOI on the soil oxygen content

Soil oxygen content (SO) increased with the increase of DO in irrigation water in both seasons (O3 > O2 > O1 > CK), and compared with the CK treatment, SO increased by 33.21% and 35.33% in spring–summer and autumn–winter O3, respectively, and the effect of DO in irrigation water on SO was highly significant (P < 0.01) in both seasons of the experiment, and SO in all treatments increased with the increase of DO in irrigation water, indicating that increasing the DO of irrigation water can significantly increase the SO to some extent. This is mainly because before the fruit cucumber planting, the soil was tilled, which increased the SO, and after the fruit cucumber transplanting, the implementation of MNOI began, although the root system developed to different degrees at each stage of growth, soil respiration intensity varies, and the soil oxygen consumed varies, but, with the increase of the crop water demand intensity, the frequency of oxygen-enhanced irrigation also increased, plus oxygen-enhanced irrigation also in the whole reproductive period, however, as the intensity of crop water demand increased, the frequency of oxygen-enhanced irrigation increased, and the oxygen-enhanced irrigation was also carried out continuously throughout the reproductive period. When the soil oxygen content was below 9% to 10%, the crop root development was affected, and most of the crop roots would stop developing when the soil oxygen content was below 5%. In this study, the SO was between 10 and 20% during the whole reproductive period, which is suitable for root growth and development. In addition, DO is related to many factors; changes in meteorological parameters, water temperature, water depth, and solutes in water will cause changes^54^, and changes in soil texture, temperature, and humidity will also affect the changes in SO.

**Figure 8:**
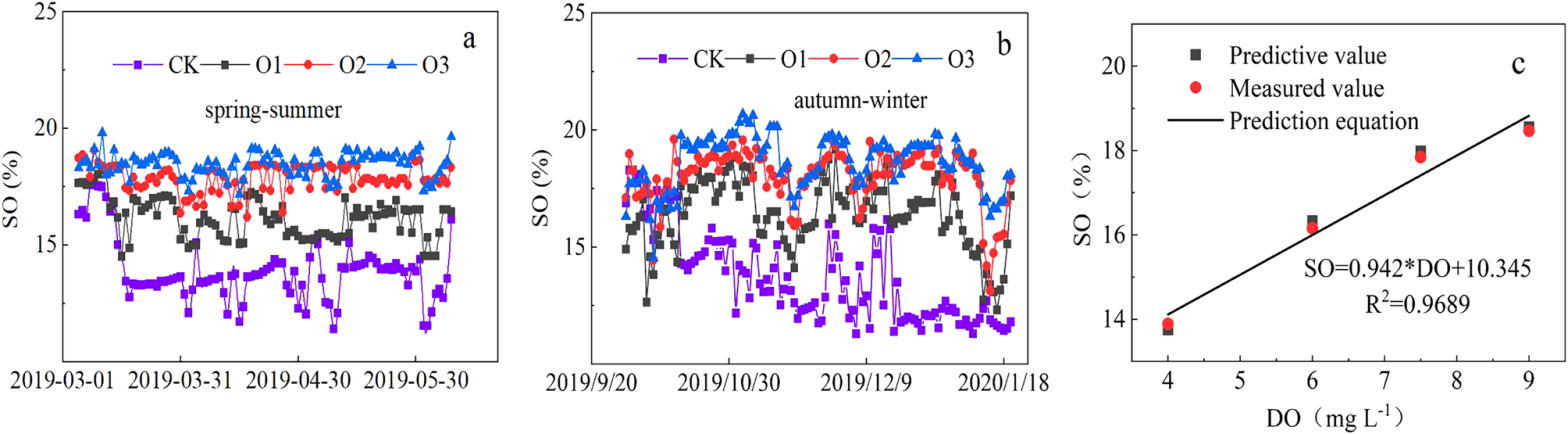
Changes of SO in spring–summer (**a**) and autumn–winter (**b**), and relationships between SO and DO in irrigation water (**c**).

Based on the real-time monitoring data of soil oxygen in the spring–summer of fruit cucumber in 2019, the model of dissolved oxygen (DO) in irrigation water and soil oxygen content (SO) under this test condition was established (Fig. 8c): SOC = 0.942*DO + 10.345 (R^2^ = 0.9689), and the data of soil oxygen content measured in autumn–winter of 2019 were verified, and the measured values were compared with predicted values were subjected to error analysis, and the average relative error was 2% (less than 5%), indicating that the linear equation is reliable and has a good predictive effect.

## Discussion

In this study, MNOI not only replenished water nutrients to the crop root zone, but also achieved root zone oxygenation to improve the gas environment in the root zone (SO, redox potential, aerated porosity, and soil oxygen diffusion rate), and SO increased by 33.21% and 35.33% in spring–summer and fall-winter O3 treatments, respectively, compared to CK treatment (Fig. 8a and b), indicating that oxygenated irrigation significantly increased SO and permeability. This result is consistent with that of Wang^38^, but it is different for the study object (cotton) and the study area. Oxygen-enriched irrigation water, which can carry dissolved oxygen into the soil, is an effective way to improve the gaseous environment in the soil root zone^55,56^.

Root respiration is one of the indicators to assess root and crop growth^57^, first of all, an improved gas environment in the root zone promotes root growth and soil respiration, which facilitates the development of root morphology^58^, in addition, dissolved oxygen affects soil microbial and enzyme activities^7,59^. In this study, compared with CK, the soil respiration rate of spring–summer and autumn–winter O3 increased by 55.22% and 110.34%, respectively, while enriching the number of soil microorganisms such as bacteria, fungi, and actinomycetes (Fig. 7a), and was conducive to the reproduction and growth of microorganisms, the rapid decomposition of humus, the release of more nutrients for plant absorption and utilization, and further improved the crop root-soil environment. Secondly, the improved gas environment in the root zone also increases the activities of soil urease, AP, catalase and MC/MN (Fig. 7b and c), which in turn enhances the uptake of soil nutrients (such as nitrogen, phosphorus, potassium, organic matter, etc.) by the root system and further strengthens the root system, which in turn can draw more water and nutrients from the soil for growth and development. Zhao et al.^60^ also stated that increasing the DO concentration of irrigation water promoted crop root growth and improved water and nutrient uptake. Increasing inter-root aeration increased soil enzyme activity and promoted the conversion efficiency of soil N and organic matter, thus increasing nitrogen uptake^61^. However, in this study, the differences in soil bacteria, fungi, actinomycetes, and soil urease were greater between the two seasons, probably due to the lower temperatures and lack of light and heat in autumn–winter resulting in lower microbial load and enzyme activity.

The growth of the root system provides a sufficient substrate for the crop to carry out photosynthesis, promotes plant growth and increases the LAI, which facilitates chlorophyll accumulation^62,63^ and guarantees smooth photosynthesis and DMA^64^. In this study, the LAI was higher in both spring–summer than in autumn–winter, which was due to the abundant light and heat in spring–summer, while the cold weather in autumn–winter restricted the growth of fruit cucumbers to some extent. Compared to CK, the A and DMA of O3 increased by 44.90% (35.00%) and 49.81% (127.25%), respectively (Figs. 3 and 5), and yield and IWUE increased by 22.47% (28.04%) and 22.39% (28.05%), respectively, in the spring–summer (autumn–winter), and fruit cucumber yield and quality, as well as economic efficiency, were improved (Figs. 4 and 6). This is consistent with the results of previous studies^63,65^, but there is a difference between the two methods of oxygenation. The results of Niu, et al.^13^ showed that oxygenation by subsurface drip irrigation improved irrigation water use efficiency and yield, but the irrigation method in this study was based on sub-membrane drip irrigation, which is consistent with the results of this study. In addition, in this study, the fruit quality in autumn–winter was lower than that in spring–summer, especially, the fruit vitamin C in autumn–winter was higher than that in spring–summer (Fig. 4), which was mainly due to sufficient heat from light in spring and summer, which was favorable to fruit quality improvement, but not to the accumulation of vitamin C, which was easily decomposed under sufficient light conditions, while vitamin C was closely related to climatic conditions, fruit picking time, and testing time, etc. The explanation of the mechanism on fruit quality needs to be further explored. The mechanism explanation for fruit quality needs to be further explored.

## Conclusion

MNOI increased soil oxygen content, improved soil enzyme activity, enriched soil microorganisms, promoted fruit cucumber growth (plant height, LAI), increased chlorophyll content, enhanced A, and facilitated plant DMA. Fruit cucumber yield and quality increased with increasing dissolved oxygen in irrigation water (dissolved oxygen increased from 4 to 9 mg L^−1^), the better level was a high level of dissolved oxygen in irrigation water O3 (9.0 mg L^−1^).

A model of dissolved oxygen (DO) in irrigation water and soil oxygen content (SO) was established under the conditions of this experiment: SO = 0.942*DO + 10.345 (R^2^ = 0.9689) with an average relative error of 2% (less than 5%).

## Data Availability

The datasets used and analysed during the current study available from the corresponding author on reasonable request.
